# Precise determination of toll-like receptor (TLR) 7 expression in multiple human tumor types

**DOI:** 10.1186/2051-1426-2-S3-P265

**Published:** 2014-11-06

**Authors:** Marie Cumberbatch, Nicola Haughton, Emily Foster, Xiu Huan Yap, Simon Barry, Setsuko Yamamoto, Masashi Murata, Robert W Wilkinson, Christopher Womack

**Affiliations:** 1 AstraZeneca, United Kingdom; 2 Sumitomo Dainippon Pharma, United Kingdom; 3 MedImmune, United Kingdom

## 

TLR7 agonists are being progressed as potential immunotherapeutics for the treatment of cancer. TLR7 agonism is believed to trigger a plasmacytoid dendritic cell driven immune response which drives anti-tumor efficacy. In addition to modulation of the immune system, TLR7 may be involved in tumor progression due to expression of TLR7 by tumor cells themselves [1]. To determine the precise distribution of TLR7 in different tumor types, a comprehensive validation of anti-TLR7 antibodies was conducted by immuohistochemistry (IHC). One antibody (Epitomics, 3923-1), out of five selected from scientific citations, proved specific for TLR7 following analyses by western blot of cell lysates, and by IHC of formalin fixed paraffin embedded (FFPE) cell pellets, prepared from HEK293 cells either stably transfected, mock-transfected or non-transfected with TLR7. 3923-1 was validated further across human spleen, lymph node and tonsil, revealing the expected tissue and cellular localization for these lymphoid organs. Assessment of TLR7 expression in a selection of tumor samples revealed non-specific tumor cell staining for the rejected antibodies, compared with minimal tumor staining for 3923-1. To explore further the distribution of TLR7, 5 tissue microarrays comprising 18 different human tumor types (6-25 patients/tumor type, triplicate cores) and 14 normal tissues (5 donors/tissue type, duplicate cores) were examined by IHC using 3923-1. Staining for TLR7 was scored by a pathologist (4-point scale: 0 negative, 1+ weak, 2+ moderate, 3+ strong) for tumor and immune cell compartments. Out of 18 tumor types examined, 5 were negative for tumor cell expression of TLR7 (ovarian, glioma, thyroid, liver, renal) and 9 exhibited a proportion (4%-36%) of patients with weak staining (breast, lung, colorectal, pancreatic, gastric, head & neck, melanoma, esophageal, endometrial). Moderate staining was observed for 11%-17% of sarcoma, prostate and bladder tumors. Corresponding normal tissue epithelium was largely negative for TLR7. Importantly, an increased density of immune infiltrates was observed in tumor tissues compared with normal tissues, and a greater proportion of the immune infiltrates were TLR7 positive. These data demonstrate that TLR7 may be less frequently expressed by tumor cells than suggested by the literature and that all tumor types exhibit a marked TLR7 positive immune cell infiltrate. Together, these data identify tumour types that might benefit from TLR7 therapy and may guide patient selection.
